# Comparative analysis of the physical properties of murine and human S100A7: Insight into why zinc piracy is mediated by human but not murine S100A7

**DOI:** 10.1016/j.jbc.2023.105292

**Published:** 2023-09-26

**Authors:** Simone A. Harrison, Anais Naretto, Swati Balakrishnan, Yasiru R. Perera, Walter J. Chazin

**Affiliations:** Departments of Biochemistry and Chemistry, and Center for Structural Biology, Vanderbilt University, Nashville, Tennessee, USA

**Keywords:** S100 proteins, EF-hand protein, fluorescence, Ca^2+^-binding protein, nuclear magnetic resonance, Zn^2+^ binding protein, crystal structure

## Abstract

S100 proteins are a subfamily of EF-hand calcium-binding proteins found primarily in vertebrate animals. They are distinguished by binding of transition metals and functioning in both the intracellular and extracellular milieu. S100A7 functions in the protection of the skin and mucous membranes and is a biomarker in inflammatory skin disease. A recent study of *Neisseria gonorrhoeae* infection revealed that human but not murine S100A7 could be used to evade host nutritional immunity. To understand the molecular basis for this difference, we carried out a comparative analysis of the physical and structural properties of human and murine S100A7. The X-ray crystal structure of Ca^2+^-loaded mouse S100A7 (mS100A7) was determined to 1.69 Å resolution, and Ca^2+^-induced conformational changes were assessed by NMR. Unlike human S100A7 (hS100A7), which exhibits conformational changes in response to binding of Ca^2+^, no significant changes in mS100A7 were detected. Dynamic light scattering, circular dichroism, and a competition chelator assay were used to compare the Zn^2+^ affinity and the effects of ion binding on mS100A7 *versus* hS100A7. Alignment of their sequences revealed a substantial difference in the C-terminal region, which is an important mediator of protein–protein interactions, suggesting a rationale for the specificity of *N. gonorrhoeae* for hS100A7. These data, along with more detailed analysis of S100A7 sequence conservation across different species, support the proposal that, although hS100A7 is highly conserved in many mammals, the murine protein is a distinct ortholog. Our results highlight the potential limitations of using mouse models for studying bacterial infections in humans.

S100A7 is one of the 24 members of the S100 subfamily of EF-hand Ca^2+^-binding proteins, which are found primarily in vertebrate animals (1). The distribution of S100 proteins is tissue- and cell type-specific, and they are believed to be specialized Ca^2+^ sensors with distinct roles from the ubiquitous intracellular Ca^2+^ sensor calmodulin and related proteins. S100 proteins are also unique because many function in the extracellular milieu (2). S100A7 was first identified from its role in inflammation in psoriatic lesions and was termed psoriasin (3). Subsequent studies showed it is overexpressed in different skin diseases including atopic dermatitis and skin cancer (4). Human S100A7 is most well characterized for its role in immune system defense against bacteria in the skin and mucous membranes. Multiple mechanisms for this activity have been proposed, including nutritional immunity *via* sequestration of zinc, cross-linking to other proteins in the wound environment, and direct adherence to bacteria (5).

S100 proteins are comprised of two EF-hand helix-loop-helix Ca^2+^-binding motifs. They are distinguished from other EF-hand proteins by a unique, noncanonical S100-specific Ca^2+^ binding loop in the N-terminal EF-hand, an intrinsic dimeric architecture, and the binding of Zn^2+^ and other transition metals at the dimer interface. The transition metal binding sites are largely preformed (6), but the modest conformational changes induced upon transition metal binding do lead to important functional outcomes (7).

The binding of Zn^2+^ has been shown to be a central factor in the antimicrobial activity of S100A7; sequestration of Zn^2+^ by S100A7 starves invading pathogens of this essential metal, a mechanism termed nutritional immunity (8). Remarkably, *Neisseria* *gonorrhoeae* has evolved the ability to utilize human S100A7 (hS100A7) as a Zn^2+^ source through a zinc piracy mechanism (9). However, a surprisingly large difference in the effect of hS100A7 and mouse S100A7 (mS100A7) on the growth of *Neisseria gonorrhoeae* has been reported; the murine protein is unable to mediate zinc piracy (10). This was particularly surprising in light of the wide use of transgenic mouse models to investigate human S100A7 function, particularly as models for bacterial infections in humans. Murine S100A7 is considered to have similar roles as human S100A7 because they have similar expression patterns associated with similar phenotypes (11). However, human and murine S100A7 have only a limited level of sequence similarity considering they are classified as homologs (31.7% identical, ∼50% conserved) (Fig. 1). Questions about the evolution and conservation of S100 genes, including S100A7, have been raised for more than 20 years (12).

**Figure 1:**
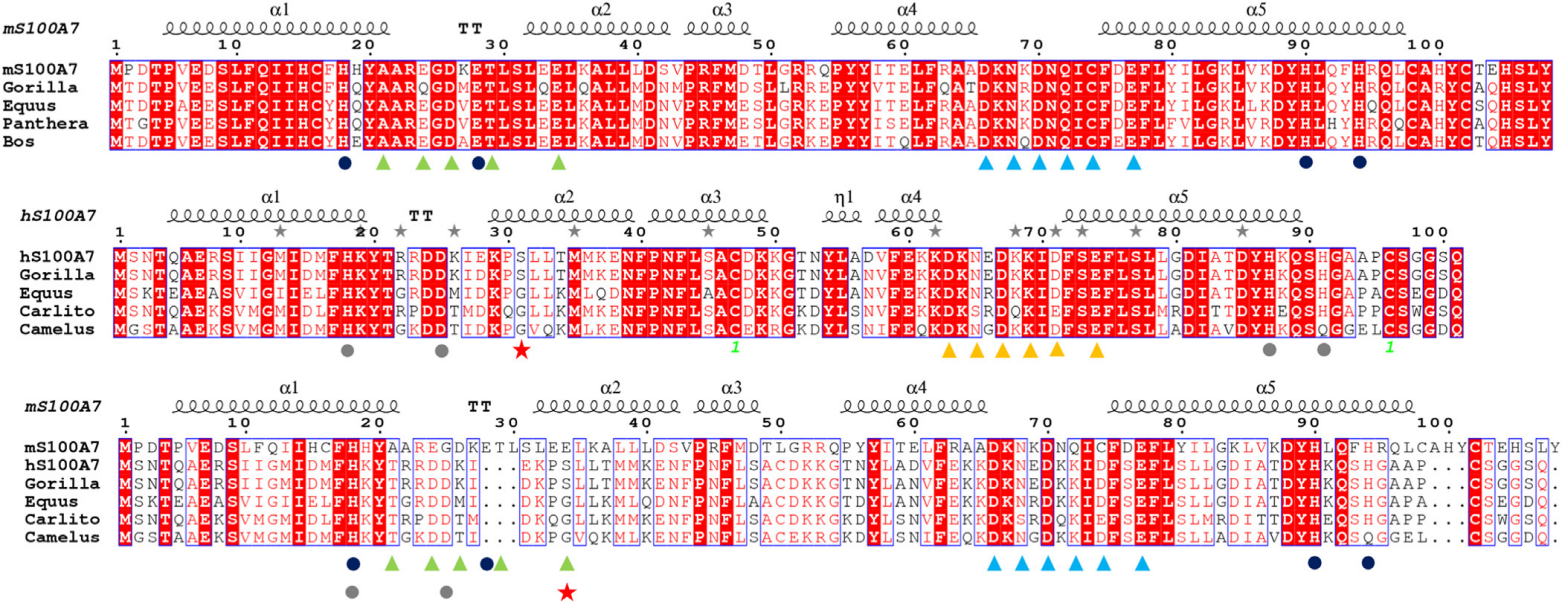
**Sequence alignment of mS100A7 and hS100A7.** (*Top panel*) mS100A7 alignment with a selection of homologs. *Dark blue dots* indicate conserved residues in the Zn^2+^ binding site. The *green and light blue triangles* indicate conserved residues of the N- and C-terminal EF-hand Ca^2+^ binding loops, respectively. The sequence with the lowest identity (79.6%) is *Panthera pardus*. (*Middle panel*) Alignment of hS100A7 with a selection of homologs. *Gray dots* indicate conserved residues in the Zn^2+^ binding site. *Orange triangles* indicate conserved residues in the C-terminal EF-hand Ca^2+^ binding loop. The sequence with the lowest identity (69.3%) is *Camelus ferus*. (*Bottom panel*) comparison of mS100A7 to hS100A7 and its homologs. The sequence identity between the human and murine proteins is 31.7%. hS100A7 and its homologs are characterized by a deletion in the N-terminal EF-hand, and mS100A7 and its homologs are distinguished by a longer and conserved C-terminal tail. *Red stars* identify the position where the strictly conserved bidentate Glu required for high affinity Ca^2+^ binding is substituted in hS100A7 and its homologs. hS100A7, human S100A7; mS100A7, mouse S100A7.

Given the central role of zinc binding in the antimicrobial function of S100A7 and the proliferation of transgenic mouse models, we set out to determine to what extent the physical properties of these two proteins are different. Human S100A7 has been extensively characterized; Ca^2+^ and Zn^2+^ affinities have been measured, and X-ray crystal structures have been determined in the Ca^2+^ and Zn^2+^-loaded state (13). Circular dichroism (CD) studies show a reorganization of helices upon binding of either zinc or calcium but no significant further change upon binding of both ions (14). Human S100A7 has an internal disulfide bond that is understood to be important for Zn^2+^ binding and function because the oxidized protein is better able to sequester Zn^2+^ (15). In contrast, there are no reported studies of the zinc and calcium binding properties or crystal structures of mS100A7.

Here, we report the development of a protocol to produce mg quantities of recombinant mS100A7 and the determination of its three-dimensional structure by X-ray crystallography. We also used a combination of biophysical methods in solution to characterize the effects of binding Ca^2+^, Zn^2+^, and both ions on the structure of the protein and to measure Zn^2+^ affinity and stoichiometry. Detailed comparison of human and murine S100A7 shows that they have some distinct physical properties. These data combined with detailed sequence analysis supports the proposal that human and murine S100A7 are distinct orthologs and the mouse protein has been mislabeled as S100A7, consistent with the species specificity of *N. gonorrhoeae* for hS100A7 upon infecting its host.

## Results

### Murine S100A7 has a stronger tendency to aggregate than human S100A7

Initial attempts at purifying murine S100A7 were based on published protocols for human S100A7, which is routinely expressed in *E. coli* BL21(DE3) and purified using a standard chromatographic strategy of ion exchange and size-exclusion chromatography (16). However, the isoelectric point of mS100A7 is 5.7, and the murine protein is poorly soluble below pH 7, so we had to adopt a different approach. This was based on purification at higher pH (8.0) combined with anion exchange chromatography (17), although there remained a significant level of impurities, so modifications to this protocol were required as described in Experimental procedures.

In the course of developing the purification protocol, we observed that although S100 proteins in general are known for their stability and solubility, mS100A7 is not very soluble over ∼5 mg/ml, and a variety of conditions can cause it to aggregate and precipitate. Moreover, mS100A7 precipitates at NaCl concentrations outside the range 50 to 250 mM. We also found that mS100A7 begins to aggregate within 24 h of exposure to Ca^2+^, especially at protein concentrations of 100 μM or above. These observations contrast sharply with the high solubility of hS100A7 in a range of experimental conditions.

To further characterize the biophysical properties of mS100A7, we investigated its thermal denaturation by monitoring the temperature dependence of CD at 222 nm. Although a gradual melting of the helical secondary structure is observed, the reduction in the peak minimum at 222 nm is only ∼50% complete even at 110 °C (Fig. S1). Hence, like many S100 proteins, mS100A7 is extremely stable, remaining at least partially folded even in boiling water/buffer. Notably, this observation supports the addition of a heating step in the purification protocol as described in Experimental procedures.

A number of S100 proteins undergo transitions from the intrinsic dimeric state to higher order oligomers upon binding calcium (18). Dynamic light scattering was used to examine the state of oligomerization of mS100A7 in the absence and presence of Ca^2+^ and compare to hS100A7. The results showed there is no difference in the hydrodynamic radius upon binding of Ca^2+^ for either the murine or human proteins (Table S1).

### Murine S100A7 binds zinc with an affinity similar to human S100A7

To determine if the difference in the effect of mS100A7 and hS100A7 on bacterial growth was the result of reduced zinc binding, we set out to compare the affinity and stoichiometry of Zn^2+^ for both proteins using a competition chelator assay. A previously described protocol from the Nolan laboratory (15) using the fluorescent zinc chelator Zinpyr-4 (ZP4) (K_d_ = 0.65 nM) was adapted for this purpose; a concentration-dependent increase in fluorescence signal is fit based on the protein competing with the chelator for Zn^2+^. Due to the propensity of mS100A7 to precipitate, instead of progressively titrating zinc into a single mixture of ZP4 and protein as reported previously, we prepared individual samples of hS100A7 or mS100A7 and ZP4, to which was added different concentrations of Zn^2+^.

**Figure 2:**
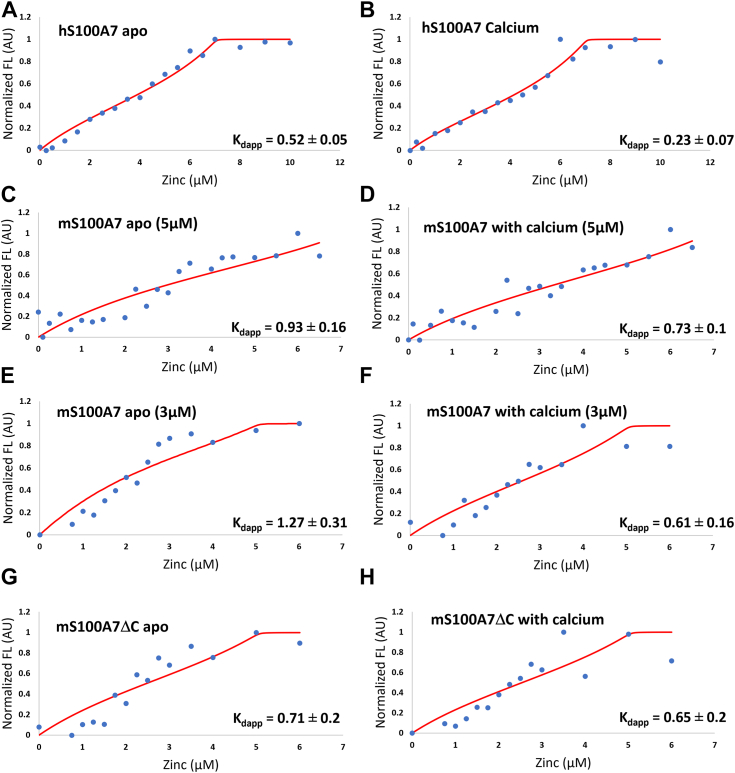
**Determination of zinc binding affinity of hS100A7 and mS100A7 by a competition chelator approach.** The Zn^2+^-induced response measured by monitoring the fluorescence signal from 2 μM ZP4 in the presence of hS100A7, mS100A7, and mS100A7ΔC. The plots of the data and fits shown are the individual titrations closest to the mean selected from the triplicate measurements (Fig. S4). *A*–*F*, titrations performed with 5 μM hS100A7 in the absence (*A*) and presence of 25 μM Ca^2+^ (*B*); 5 μM mS100A7 in the absence (*C*) and presence of 25 μM Ca^2+^ (*D*); 3 μM mS100A7 in the absence (*E*) and presence of 15 μM Ca^2+^(*F*). *G* and *H*, 3 μM mS100A7ΔC in the absence (*G*) and presence of 15 μM of Ca^2+^ (*H*). The calculated apparent K_d_ values in nM units are listed at the *bottom right* in each plot. All data were fit to the two equivalent binding sites per dimer model. The *red line* shows the fit of the data calculated in DynaFit. hS100A7, human S100A7; mS100A7, mouse S100A7; mS100A7ΔC, mouse S100A7 tailless; ZP4, Zinpyr-4.

Figure 2 shows plots of the data and fits to the binding curve for both hS100A7 and mS100A7. Since S100A7 is homodimeric with two symmetrically disposed, equivalent binding sites at the dimer interface, we used a ‘two equivalent sites per dimer’ model to fit the data. The fits yielded apparent K_d_ values of 0.70 ± 0.11 nM in absence and 0.31 ± 0.07 nM in presence of Ca^2+^, respectively, similar to the previously reported values of 0.43 nM and 0.58 nM (15). Having established the protocol with the human protein, the approach was then applied to mS100A7. We note the mS100A7 data were even noisier than that obtained for hS100A7, presumably a by-product of the greater tendency of mS100A7 to aggregate. The data were fit using the same model as for hS100A7, yielding apparent K_d_ values of 0.85 ± 0.17 nM and 0.72 ± 0.12 nM in absence and presence of calcium, respectively. These results show that the human and mouse proteins do not significantly differ in their ability to bind zinc with high affinity.

### The structure of Ca^2+^-loaded mS100A7 is more open than hS100A7

Multiple X-ray crystal structures of hS100A7 have been determined (PDBID: 1PSR, 4AQJ, 2WOR) (13, 19, 20). However, despite extensive screening around the conditions for crystallization of hS100A7 and then a number of broad screens, all attempts to crystallize mS100A7 proved unsuccessful. After careful analysis of the sequence and close examination of the NMR spectrum of mS100A7, we surmised that the last eight residues of the C terminal tail may not be structured and hypothesized that removal of these residues would promote crystallization. A crystallization trial was set up by adding a small amount of trypsin to the mother liquor used for the full-length mS100A7 protein. This produced an initial set of promising crystallization conditions.

Based on this observation and careful analysis of secondary structure predictions, a construct truncating the protein at His100 (mouse S100A7 tailless [mS100A7ΔC]) was prepared and used for subsequent crystallization trials. To ensure the truncated protein was properly folded, we compared its CD spectra in the absence and presence of Ca^2+^ and its affinity for Zn^2+^ to the corresponding data for full-length mS100A7. The CD analysis showed no significant difference from the full-length protein in the alpha helical secondary structure (Fig. S2). Due to the limited solubility of mS100A7ΔC, zinc binding data could only be obtained at a concentration of 3 μM, so these were remeasured for the full-length protein (Fig. 2, *C* and *D*). Fitting of the data yielded apparent K_d_ values in absence and presence of Ca^2+^ of 1.2 ± 0.4 nM and 0.93 ± 0.35 nM, respectively, for mS100A7ΔC and 1.1 ± 0.29 nM and 0.55 ± 0.16 nM, respectively, for full-length mS100A7. The similarity in the CD spectra and Zn^2+^ affinities support our use of the mS100A7ΔC truncation for determining the structure of the protein.

Crystallization trials performed on mS100A7ΔC yielded diffracting crystals in the presence of Ca^2+^, and the structure could be refined to 1.69 Å resolution. The crystal was orthorhombic, in space group P2_1_2_1_2_1_, and contained one homodimer per asymmetric unit (Table S3). The two protomers of the mS100A7 homodimer are very similar with a root mean square deviation (RMSD) over all Cα atoms of 0.71 Å. The structure is well-defined with the exception of the six C-terminal residues (Arg95-His100); most of the main chain of residues R95-A99 are defined in only one protomer (A) but not in the other, and the side chains for these residues had poor density and several could not be modeled.

**Figure 3:**
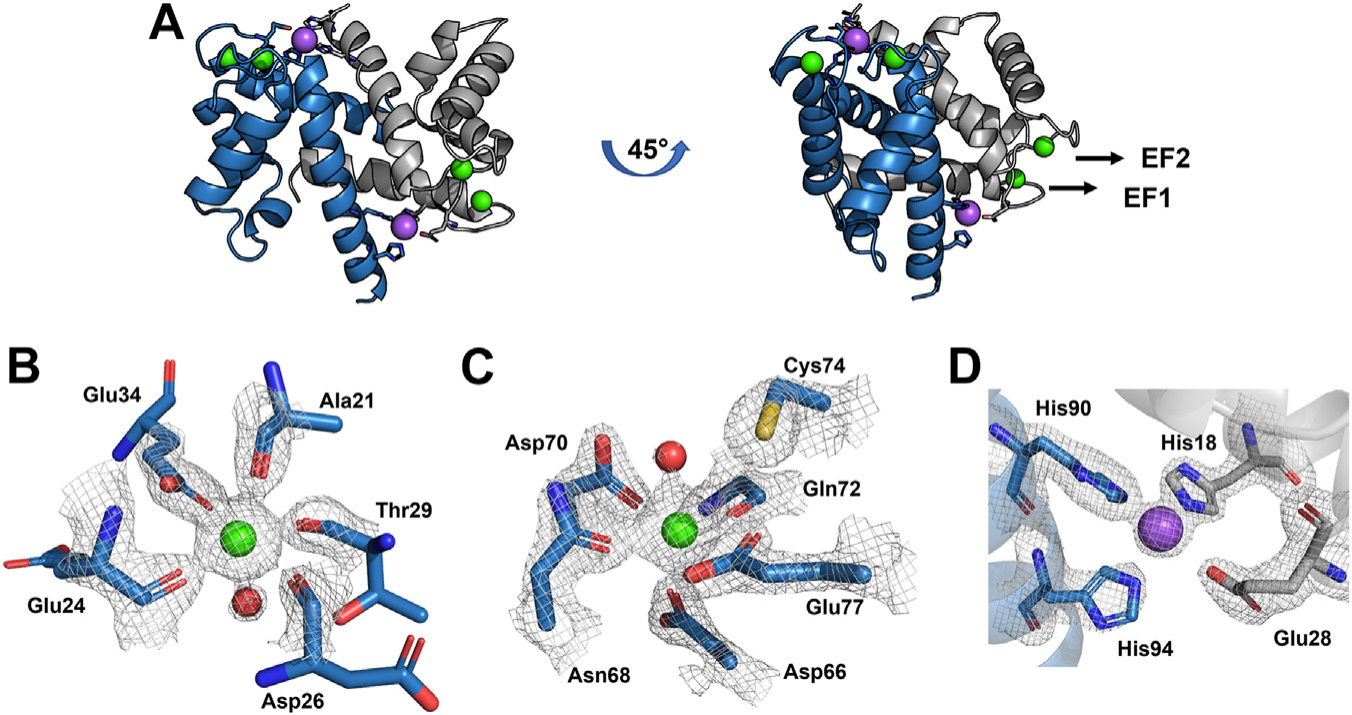
**The X-ray crystal structure of mS100A7.***A*, cartoon diagram of the structure with the four Ca^2+^ ions shown as *green spheres*. The two EF-hand Ca^2+^ binding loops are labeled in the *right panel*. *B*, close-up view of the N-terminal, S100-specific Ca^2+^-binding site. The ion is chelated by the main chain carbonyl oxygen atoms of Ala21, Glu24, Asp26, and Thr29, plus both side chain oxygen atoms of Glu34, and a water molecule (*red sphere*) stabilized by the Thr29 side chain. Electron density (2Fo-Fc) is contoured at 2 σ. *C*, close-up view of the C-terminal Ca^2+^ binding site. The ion is chelated by single side chain oxygen atoms of Asp66, Asn68, and Asp70, plus the Gln72 main chain carbonyl, both side chain oxygen atoms of Glu78, and a water molecule (*red sphere*) stabilized by the side chains of Asp70 and Cys74. Electron density (2Fo-Fc) is contoured at 2 σ. *D*, close-up view of the Zn^2+^ binding site showing the electron density for the side chains of conserved Zn^2+^ binding residues His18, Glu28, His90, and His94. A high occupancy sodium atom is displayed as a *purple sphere*. The electron density is contoured at 1σ. mS100A7, mouse S100A7.

The structure of mS100A7 revealed the canonical S100 protein dimeric architecture; each protomer is organized into two EF-hands with the hydrophobic core integrated across the dimer interface (Fig. 3*A*) (21). Like all Ca^2+^-loaded full-length S100 proteins, mS100A7 occupies an open conformation with H_III_ shifted out from the core exposing a hydrophobic cleft. One interesting feature in the structure is that the linker between the two EF-hand motifs (Val43-Gln54) is different in the two protomers with an RMSD over all Cα atoms of 2.9 Å. In protomer A, the linker forms a short α-helix from Pro44 to Asp48, which is followed by a type I β-turn at Thr49-Arg52. Moreover, within this structured linker region, Phe46-Thr49 and Gly51-Gln54 form niche4r motifs, characteristic for binding cations such as Na^+^, K^+^, Ca^2+^, and Mg^2+^ (22). The linker in protomer B is remarkably different with no identifiable motifs, presumably due to the crystal packing.

The N-terminal S100-specific EF-hand (EF1) is formed by helices H_I_ (Pro5-Ala22) and H_II_ (Ser31-Ser42) with a 14-residue Ca^2+^ binding loop (Ala21-Glu34) between them (23, 24). The Ca^2+^ ion is coordinated in a canonical pentagonal bipyramidal geometry by the backbone carbonyl oxygen atoms of Ala21, Glu24, Asp26, and Thr29, plus the critical, highly conserved bidentate carboxylate of Glu34 and a structural water molecule stabilized by an H-bond to Thr29 Oγ1 (Fig. 3*B*). The role of the Thr29 side chain is unique as the water molecule in the coordination sphere is ordinarily stabilized by the side chain of the ninth residue in the loop, which in this case is Ser31 (2). Interestingly, in this structure, Thr29 Oγ1 is also engaged in stabilizing the EF-Hand, forming an H-bond with the carboxylate of the Glu24.

The C-terminal EF hand is formed by helices H_III_ (Tyr57-Ala65) and H_IV_ (Asp76-Leu97) with a canonical 12-residue Ca^2+^ binding loop (Asp66-Glu77) between them. The Ca^2+^ ion is coordinated by side chain oxygen atoms of Asp66, Asn68, and Asp70, plus the backbone carbonyl oxygen of Gln72, the critical, highly conserved bidentate Glu77, and a water molecule stabilized by a bifurcated H-bond with Asp70 Oδ and Cys74 SH (Fig. 3*C*). The Ca^2+^ coordination geometry in canonical EF-hands is pentagonal bipyramidal (24), but in mS100A7, it is more octahedral.

The two symmetrically disposed canonical Zn^2+^ binding sites at the dimer interface, composed of the side chains of His18 and Glu28 from one chain and His 90 and His94 from the other, are all visible in the structure (Fig. 3, *A* and *D*). However, the density is not particularly well-defined, presumably due to the absence of a bound Zn^2+^ ion. Nevertheless, the overall geometry of these sites is set up for tetrahedral coordination, and in fact, a Na^+^ ion is found in both sites. A dataset was recorded close to the K-edge of zinc (1.28 Å), but no anomalous signal was found during the data analysis, confirming the absence of Zn^2+^ and consistent with a Na^+^ ion being present in both sites.

**Figure 4:**
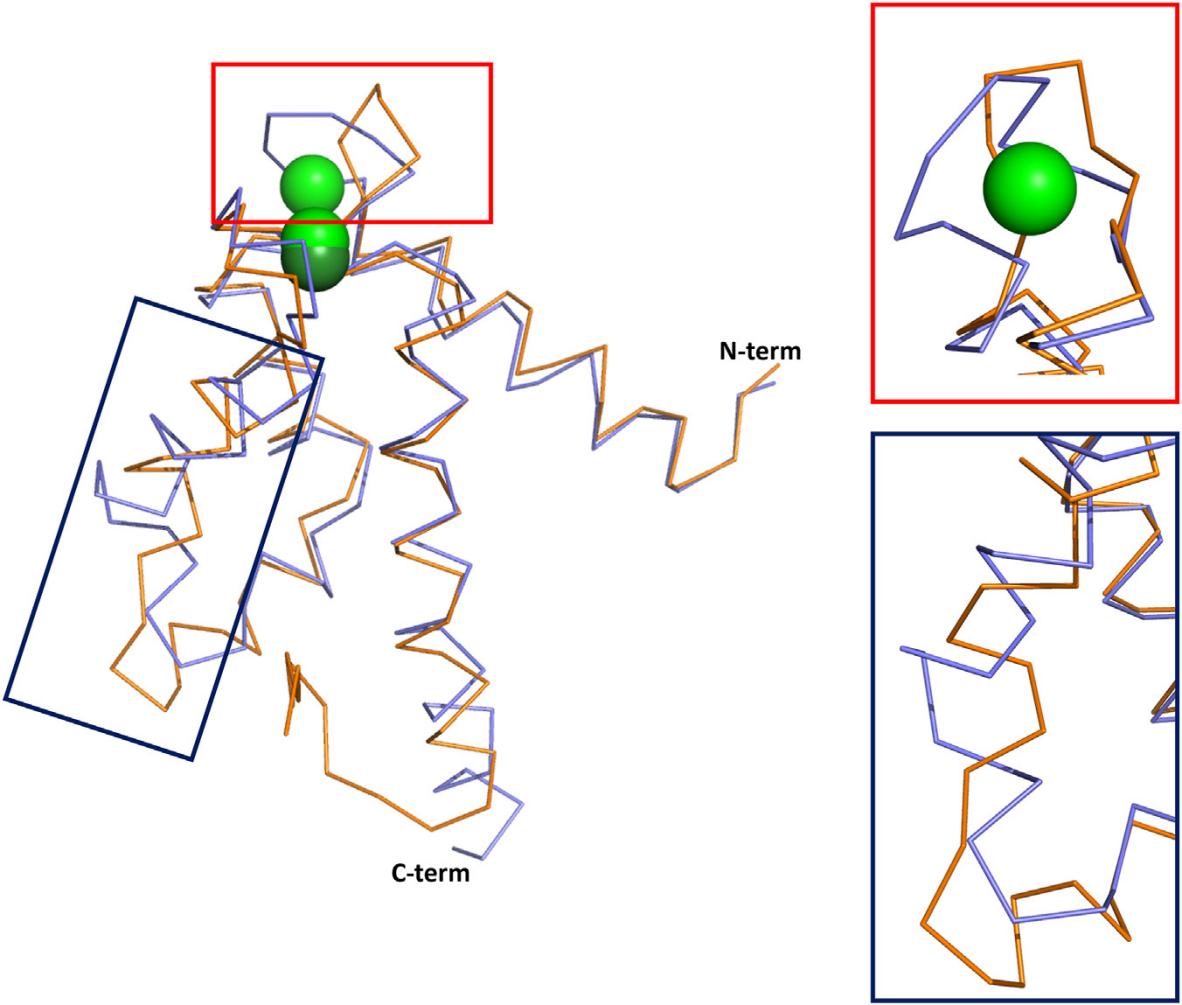
**Comparison of the structures of hS100A7 and mS100A7.***Stick* diagrams of the backbones of hS100A7 (*orange*) and mS100A7ΔC (*blue*) aligned by best fit superposition of all backbone atoms. Ca^2+^ ions are shown as *bright green spheres*, and the Ho^+^ ion in the hS100A7 structure is shown as a *dark green sphere*. The insets show regions of the structures that have the largest conformational differences. hS100A7, human S100A7; mS100A7, mouse S100A7.

Comparison of the mS100A7 structure to hS100A7 revealed that although both have the classic S100 protein homodimeric architecture, the RMSD of 1.68 Å over all Cα atoms is larger than expected for two homologs such as human and bovine S100 B, for which pairwise comparisons of human against bovine S100 B yield Cα RMSDs of 0.34 to 0.47 Å for the different human S100 B structures (PDBID: 3D0Y, 3IQO, 3CR2) (Fig. 4). The H_II_/H_III_, H_III_/H_IV_, H_I_/H_I’_, and H_IV_/H_IV’_ interhelical angles of mS100A7 indicate that the protein occupies an open conformation. Comparison of the mS100A7 and hS100A7 shows the murine protein has larger interhelical angles (Table S2), which indicates a more open conformation. Helix III is notably different between the two proteins, with mS100A7 having a more regular structure than hS100A7. The most significant difference between the two protein structures is due to mS100A7 having a standard N-terminal S100-specific Ca^2+^-binding loop (Ala21-Glu34), whereas this loop in hS100A7 is truncated to 12 residues (Tyr19-Ser30). The shorter length combined with the absence of the strictly conserved bidentate Glu residue at the C terminus of the loop precludes binding of Ca^2+^ ions with appreciable affinity to this loop in human S100A7. A second difference between the two S100A7 proteins with structural implications is the length and arrangement of the linkers between the two EF-hands. The linker for mS100A7 (Val43–Ala53) is four residues shorter than hS100A7 (Phe39-Tyr53) and packed differently, which is reflected in the high value of RMSD between the linkers (3.37 Å over all linker Cα atoms) (Fig. 4). Overall, the extent of the difference in the structures of mS100A7 and hS100A7 was initially puzzling as they are greater than would be expected for two homologs, such murine and human S100A9 whose corresponding RMSD is 0.92 Å (PDBID: 6ZDY, 1IRJ).

### Calcium-free mS100A7 occupies an open conformation

**Figure 5:**
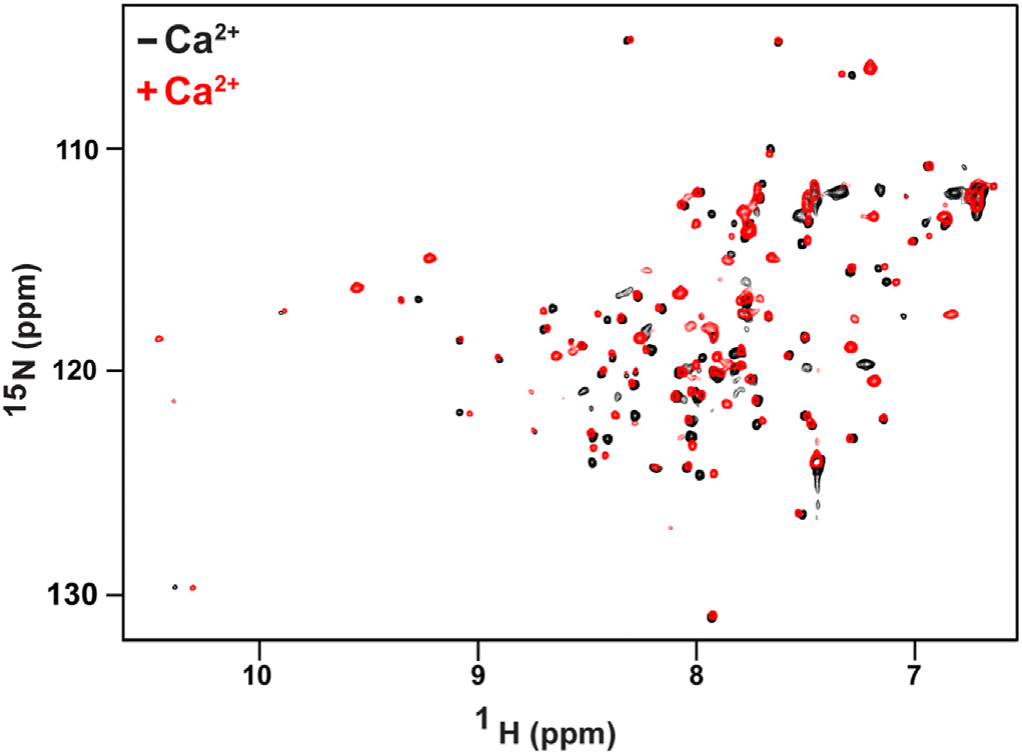
**The absence of Ca**^**2+**^**-induced conformational change in mS100A7.** Overlay of the 800 MHz ^15^N-^1^H HSQC NMR spectra obtained for 100 μM ^15^N-enriched mS100A7 obtained in the absence (*black*) and presence of 1 mM Ca^2+^ (*red*). Note that the intensity of the signals in the presence of Ca^2+^ are less uniform, which may indicate a tendency to aggregate or conformational heterogeneity. Although new signals appear in the low field region of the spectrum for the Ca^2+^-loaded state characteristic of the residues that chelate the Ca^2+^ ions, the absence of substantial changes in the chemical shifts of other residues indicate there are no fundamental changes in the conformation of the protein. HSQC, heteronuclear single quantum coherence; mS100A7, mouse S100A7.

Due to the inability to crystallize mS100A7 in the absence of Ca^2+^, we turned to solution NMR spectroscopy, a powerful means to characterize structural changes as experimental conditions are varied, to define the extent of conformational changes induced in mS100A7 by the binding of Ca^2+^. We and others have shown this approach is ideal for investigating conformational changes induced by binding of Ca^2+^ ions (25, 26). To this end, samples of ^15^N-enriched mS100A7 were prepared, and 2D ^15^N-^1^H heteronuclear single quantum coherence NMR spectra were acquired in the absence and presence of Ca^2+^ (Fig. 5). The spectra exhibit the favorable spectral characteristics observed for nearly all S100 proteins, with wide dispersion of signals and relatively narrow line widths, indicative of globular structures in both states.

Surprisingly, there is a dearth of changes in the spectrum of mS100A7 induced by the binding of Ca^2+^, less dramatic than those typically observed for other S100 proteins, for example hS100A7 (*cf.* Figs. 5 and S3). The lack of substantial Ca^2+^-induced conformational changes is highly unusual for an EF-hand calcium binding protein. Some EF-hand proteins bind Ca^2+^ so tightly that it is extremely difficult to isolate the Ca^2+^-free protein. To confirm that our Ca^2+^-free protein was truly free of Ca^2+^, the amount of residual Ca^2+^ in the ‘Ca^2+^-free’ sample was measured by ICP-mass spectrometry. Less than 0.02 equivalents of Ca^2+^ was found. Together, these results indicate mS100A7 does not undergo a significant conformational change upon binding Ca^2+^. Since the X-ray crystal structure shows the Ca^2+^-loaded state is in an open conformation, we conclude that mS100A7 occupies a preformed open conformation in the absence of Ca^2+^. This is a surprising observation as there are no known examples of an isolated S100 protein that occupies a fully open conformation in the Ca^2+^-free state.

### mS100A7 and hS100A7 differ in their responses to binding of Zn^2+^

**Figure 6:**
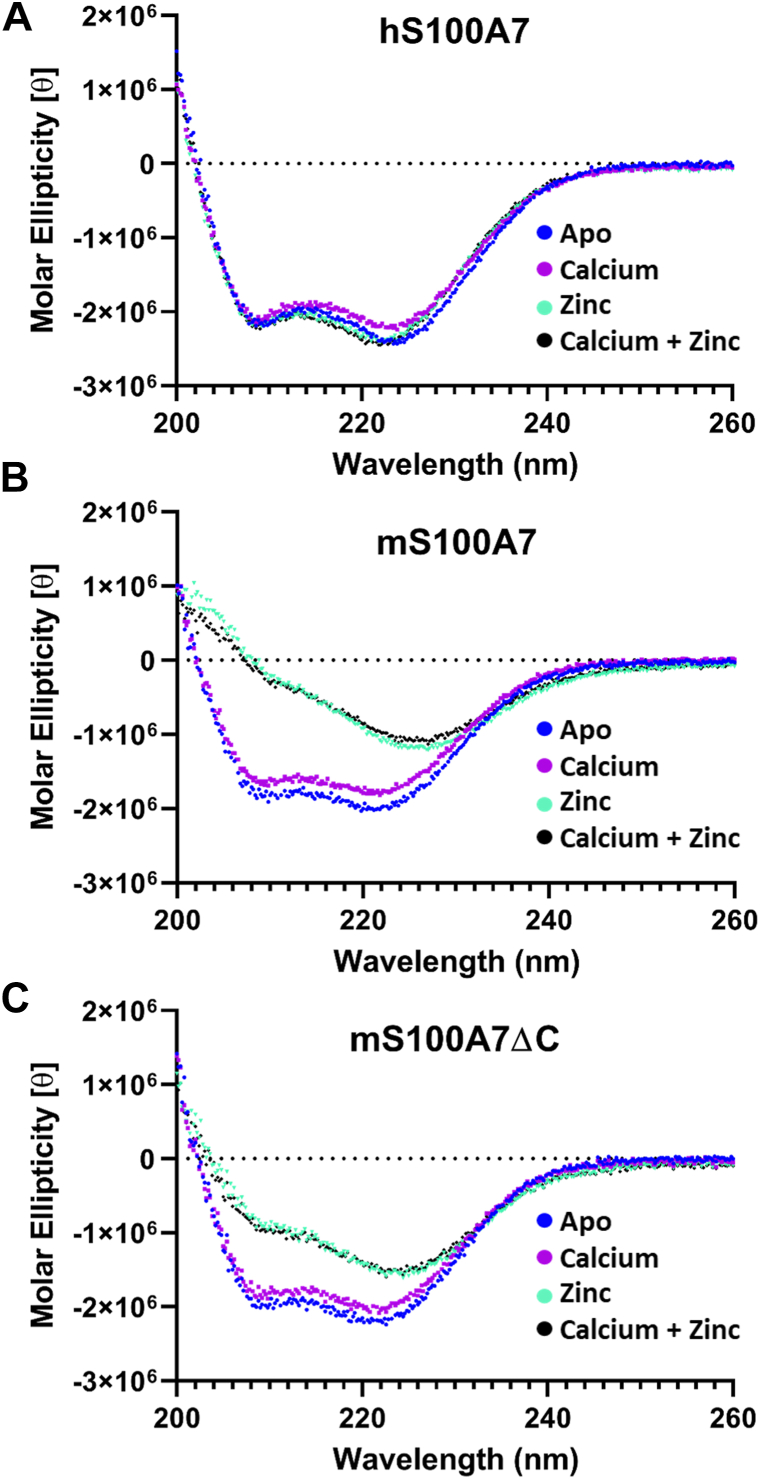
**Comparison by circular dichroism of human S100A7, murine S100A7wt, and murine S100A7ΔC.***A*, hS100A7 in 20 mM Hepes at pH 7.4 and 50 mM NaCl. *B* and *C*, mS100A7 and mS100A7ΔC in the same buffer with the addition of 1% glycerol and 1 mM TCEP. All experiments were performed at a protein concentration of 10 μM at room temperature. The Ca^2+^ spectra were obtained in the presence of a 5:1 ratio of Ca^2+^-binding site, the Zn^2+^ spectra in the presence of 2:1 ratios of Zn^2+^:protein, and the Ca^2+^+ Zn^2+^ spectra in the presence of a 5:1 ratio of Ca^2+^:binding site plus a 2:1 ratio of Zn^2+^:protein. hS100A7, human S100A7; mS100A7, mouse S100A7; mS100A7ΔC, mouse S100A7 tailless; TCEP, Tris (2-carboxyethyl) phosphine.

CD was used to probe changes in secondary structure for murine and human S100A7 upon addition of calcium and/or zinc. The far-UV CD spectra (200–260 nm) of hS100A7 and mS100A7 have the characteristic pattern of all S100 proteins with negative minima at 208 and 222 nm indicative of the dominance of helical secondary structure (Fig. 6). While the overall shape of the CD spectrum is retained, addition of calcium or zinc typically results in an increase in the negative ellipticity at 222 nm (16, 27). It is important to note that such changes arise as a result of binding-induced reorganization of the packing of helices, not an increase in helical content. For hS100A7, no significant changes in the CD spectrum were observed upon addition of Ca^2+^, Zn^2+^, or both ions (Fig. 6*A*), similar to a previous study under different conditions where only a slight increase in ellipticity was observed upon addition of either ion (14). Like hS100A7, no changes in the CD spectrum of mS100A7 were observed upon addition of Ca^2+^. However, addition of Zn^2+^ caused a substantial reduction in negative ellipticity at 208 and 222 nm (Fig. 6*B*). To understand the origin of this observation, we first ran an SDS-PAGE gel without reducing agents (Fig. S5). This showed that the change in CD ellipticity was not due to an induction of intermolecular disulfide cross-links. We then examined the distribution of oligomerization states by dynamic light scattering (Table S1). This experiment revealed Zn^2+^ induces severe aggregation of the protein, which could explain by the loss of intensity in the CD spectrum. We also characterized the change in CD induced by Zn^2+^ for the mS100A7ΔC construct lacking the eight disordered C-terminal residues and again observed the reduction in CD ellipticity, albeit not as severe as for the full-length protein (Fig. 6*C*). This suggests that the C-terminal residues of mS100A7, which are poorly conserved with hS100A7, may play a role in the Zn^2+^-induced aggregation.

## Discussion

This study was motivated by the surprisingly large difference in the effect of hS100A7 and mS100A7 on the growth of *N. gonorrhoeae* (10). We developed protocols to produce mg quantities of pure mS100A7, which enabled comparisons of the biochemical properties and structure of the two proteins. Challenges to determining the crystal structure of full-length mS100A7 were initially overcome by a limited proteolysis approach; removing residues off the flexible C-terminal tail resulted in production of crystals that diffracted to atomic resolution. Although similar overall, the most significant difference between the two proteins is in their response to the binding of Ca^2+^. Our NMR data show that mS100A7 is very unusual in that its conformation is already open in the absence of Ca^2+^, whereas it is clear that hS100A7 undergoes a substantial Ca^2+^-induced conformational change.

The structural data show there are clear differences between human and murine S100A7. However, it is unclear how this relates to differences in the ability of human but not murine S100A7 to suppress the growth of *N. gonorrhoeae*, particularly since the piracy of zinc from hS100A7 occurs in the extracellular milieu where Ca^2+^ levels are high and both proteins would be Ca^2+^-loaded. The origin of the difference in the two proteins is also not related to their ability to bind zinc as all of the transition metal binding residues are conserved (Fig. 1), and their affinities for zinc are very similar (Fig. 2).

One key difference between mS100A7 and hS100A7 is the length and sequence of their C-terminal regions (Fig. 1). This difference is particularly intriguing with respect to function in *N. gonorrhoeae* zinc piracy, for which two TonB-dependent zinc transporters TdfH and TdfJ have been implicated (9, 27). The structure of TdfH has been determined in complex with the hS100A8/S100A9 heterodimer (28), and it reveals an intimate interaction of the C-terminal region of hS100A8/S100A9 with the transporter. Moreover, as in the case of S100A7 and TdfJ, human but not murine S100A8/S100A9 serves as a zinc source for TdfH (27), and there are significant differences in the length and sequence of their C-terminal regions. If TdfH and TdfJ interact with their corresponding S100 proteins in a similar manner, the differences in the S100 C-terminal regions may well serve as a common mechanism for how *N. gonorrhoeae* selects for the human S100 proteins. This in turn supports the proposal that mouse models are not be suitable for *N. gonorrhoeae* and other bacterial infections.

Consideration of all of the data comparing mS100A7 and hS100A7 leads to questions as to whether these two proteins are functional homologs? As noted above, the level of similarity of their sequences is rather limited considering they are classified as homologs (Fig. 1) (29). Indeed, concern about the conservation of the S100A7 gene of rodents was first raised nearly 20 years ago (12). A later study suggested that the mouse and rat might have lost S100A7 genes; in contrast, S100A7 appears to be duplicated among placental mammals and led to several human S100A7 genes (30). Among the human genes are S100A7a, otherwise known as S100A15 (19, 31, 32). A recent phylogenetic analysis found vestigial traces of S100A7 and S100A15 genes for the mouse and the human, respectively, strengthening further the hypothesis of a gene loss during the evolution for both species (33). To further address the relationship between human and mouse S100A7, we conducted a search in the Mouse Genome Informatics database for other S100 proteins that were overexpressed in the skin or endothelium and could potentially function similarly to S100A7 in humans. The closest protein sequence to mS100A7 is actually mS100A9, but this gene has very low 31% sequence identity to mS100A7 and is clearly a S100A9 homolog.

These observations suggest that a portion of the genome may have been lost during evolution. Interestingly, close homologs exist for mS100A7 and mS100A15 in the gorilla and donkey genomes but not in other species such as the leopard. This fact raises the questions of how and why S100A7 genes have been deleted and duplicated, and what are the consequence of these differences at the functional and physiological level? The duplication of S100A7 genes in humans and other placental mammals, leading to new variants with unique functions and expression in distinct tissues, suggest there is a need for specialization within the S100A7 gene family. Alternatively, these different S100A7 genes may have arisen to compensate for the loss of the original/ancestral S100A15 gene.

Our biochemical and structural data, which reveal differences with respect to hS100A7, including the conformational response to binding of Ca^2+^, lead to uncertainty over whether the murine protein represents an appropriate functional analog of human S100A7 and support the proposal that mice have lost the S100A7 gene (34). To continue to use mouse models for humans, it is important to determine which S100 protein functional analogs are present and if this varies for different tissues. For bacterial infections, if indeed mS100A7 is not actually an S100A7, then the question remains as to what is the primary epithelial S100 protein in mouse? Analysis of the literature and of the Mouse Genome Informatics database does not provide sufficient clarity on this point. The development and study of ex-vivo human organoid models (35) provide a promising alternative strategy to potentially resolve this conundrum.

## Experimental procedures

### Plasmid generation

The gene of mS100A7 was synthetized by Genescript and subcloned in the pET11a plasmid using the NdeI and BamHI restriction sites. Deletion mutant mS100A7_1-100_ (mS100A7ΔC) was generated using a Q5 kit from NEB by following the manufacturer instructions. The primers used were: 5′TAAGGATCCGGCTGCTAACAAAGC 3′; 5′GTGCGCGCACAGTTGACG 3′.

### Protein overexpression

Human S100A7 was overexpressed in *E. coli* BL21(DE3) cells as described previously. In contrast, murine S100A7 only expressed well in the *E. coli* BL21 RIL cell line. In both cases, several clones were grown overnight in LB-Amp (100 μg/ml) until saturation. This preculture (10 ml) was then used the next day to inoculate 1 L of fresh LB with 50 mg ampicillin. The culture was performed at 37 °C under agitation (350 rpm) until the *A*_600_ reached approximately 0.6. Protein overexpression was induced by the addition of 0.5 mM of isopropyl β-D-1-thiogalactopyranoside. The culture was allowed to grow 4 h after induction at 37 °C prior harvest. The cells were pelleted by centrifugation using a JLA6.100 rotor, 6500 rpm at 4 °C. The pellets were transferred to a clean 50 ml falcon tube and placed at −20 °C until use. The purification of the proteins was carried out using cells from 3L cultures.

### Purification of hS100A7

The purification of hS100A7 followed a published procedure (14). Cells were resuspended in 200 ml of lysis buffer (20 mM sodium acetate at pH 5.4) using a Dounce homogenizer. The cells were disrupted using a sonicator (Fischer Model 505 Sonic Dismembrator) set at 50% power for 10 min, 5 s on 10 s off. The cellular debris were removed by centrifugation using rotor JA25.50 set at 50,000 rcf for 30 min. The supernatant was loaded onto a manually packed column of 15 ml with Source15S resin (Cytiva) previously equilibrated with the Buffer A (20 mM sodium acetate at pH 5.4) using a BioRad NGC FPLC system at 4 °C. The protein was eluted using a linear gradient over 10 column volumes generated by mixing the Buffer A and Buffer B (20 mM sodium acetate at pH 5.4, 1M NaCl). The fractions containing the protein were pooled and concentrated using an an Amicon Ultra-centrifugal Filter Unit 10-kDa molecular weight cut-off (MWCO) (Millipore-Sigma). Analysis by SDS-PAGE revealed no further purification steps were required. The protein was aliquoted, flash-frozen using liquid nitrogen, and then stored at −80 °C. Protein identity and purity were assessed by electrospray mass spectrometry.

### Purification of mS100A7 and mS100A7ΔC

Because the isoelectric point of mS100A7 is 5.7, and the murine protein is poorly soluble below pH 7, an alternate strategy for purification of hS100A7 (17) was adapted for mS100A7 and mS100A7ΔC. Protein produced with the standard protocol had a significant level of residual impurities, so an additional step was incorporated after lysing the cells. This involved heating the supernatant to 85 °C for 20 min, leveraging the intrinsically high thermal stability of S100 proteins including mS100A7.

Cells were resuspended in 100 ml of lysis buffer (20 mM Tris at pH 8.0, 100 mM NaCl, and 10 mM 2-mercaptoethanol [BME], 5% glycerol, 1 mM EDTA) using a Dounce homogenizer. The cells were disrupted using a sonicator (Fisher Model 505 Sonic Dismembrator) set at 50% power for 10 min and 5 s on/10 s off. The cellular debris was removed by centrifugation using a rotor JA25.50 set at 50,000 rcf for 30 min. The supernatant was warmed at 85 °C for 20 min, then centrifuged again using the same conditions. The lysate was then diluted with 200 ml of dilution buffer (20 mM Tris at pH 8.0, 10 mM BME) to decrease the sodium chloride concentration to ∼30 mM. The diluted supernatant was loaded onto a HiPrep DEAE 16/10 column (Cytiva) previously equilibrated with the Buffer A (20 mM Tris at pH 8.0, 50 mM NaCl, 10 mM BME, 1% glycerol) using an Akta Start (Cytiva) system at room temperature. The protein was eluted using a gradient generated by mixing the Buffer A and Buffer B (20 mM Tris at pH 8.0, 1 M NaCl, 10 mM BME, 1% glycerol). The protein-containing fractions were pooled and concentrated using an Amicon ultrafiltration system with a disc filter of 3 kDa MWCO. Higher molecular weight impurities were then removed by size-exclusion chromatography on an HiLoad Superdex S75 16/60 column equilibrated with the Buffer C (20 mM Tris at pH 8.0, 100 mM NaCl, 1 mM Tris (2-carboxyethyl) phosphine [TCEP]). The protein was concentrated using an Amicon Ultra-centrifugal Filter with 10-kDa MWCO (Millipore-Sigma) to 1 mg/ml and aliquoted in 1 ml fraction. The protein was flash-frozen using liquid nitrogen and then stored at −80 °C. Protein identity and purity were assessed SDS-PAGE and mass spectrometry.

### Preparation of metal free buffer and protein

Buffers and proteins were treated with Chelex 100, molecular grade, 200 to 400 mesh (Bio-Rad) prior to all experiments requiring addition of Ca^2+^, Zn^2+^, or both to ensure that the starting materials were metal-free. Prior to use, the resin was washed using 300 volumes of milliQ water to remove salts and any contaminants. The wet resin was then added to the buffer or the protein using plastic utensils to avoid any metal contamination. The mix was incubated overnight with slight agitation. The next day, buffers or proteins were separated from the resin by centrifugation and used immediately.

### CD spectroscopy

CD spectra were acquired using a Chirascan CD instrument (Applied Photophysics). Data were collected at 0.2 nm intervals over the range 200 to 260 nm at 25 °C using a temperature-controlled chamber. A 0.1 cm cuvette containing 180 μl of protein at 10 μM was used for all the measurements. All measurements were performed at least in triplicate, and the buffer background was subtracted for each measurement. The protein was buffer-exchanged using an Amicon Ultra-centrifugal Filter with 10-kDa MWCO (Millipore-Sigma) and then treated with Chelex resin prior to recording the spectra. For hS100A7, the buffer was 20 mM Hepes at pH 7.4 and 50 mM NaCl. For mS100A7, the buffer was 20 mM Hepes at pH 7.4, 50 mM NaCl, 1% glycerol, and 1 mM TCEP. For each measurement made in the presence of Ca^2+^ and/or Zn^2+^, the protein was incubated for 30 min in a solution containing (i) a 1:1 M ratio of CaCl_2_ for the hS100A7 or 2:1 for mS100A7; (ii) a 1:1 M ratio of ZnSO_4_; (iii) a 2:1 M ratio of ZnCl_2_; and (iv) a 2:1 M ratio of ZnCl_2_ plus a 5:1 M ratio of CaCl_2_ for hS100A7 or a 10:1 M ratio of CaCl_2_ for mS100A7. The data were processed using ProData viewer program.

### Dynamic light scattering

All experiments were performed using a DynaPro Nanostar (Wyatt) system with disposable microcuvettes (Wyatt). The protein concentration was set to 200 μM and measured after Chelex treatment. The buffer for hS100A7 was 20 mM Hepes at pH 7.4 and 100 mM NaCl. The buffer for mS100A7 was 20 mM Hepes at pH 7.4, 100 mM NaCl, 1% glycerol, and 1 mM TCEP. For the test in the presence of calcium, the protein was incubated with CaCl_2_ for 1 h prior to measurement.

### Measurement of zinc affinity

A competition chelator method using the fluorescent probe ZP4 (Santa Cruz Biotechnology) was used to measure the affinity of hS100A7, mS100A7, and mS100A7ΔC for Zn^2+^ (15). Since the method measures affinity indirectly by competition, we report apparent dissociation constants (K_dAPP_). ZP4 was dissolved in DMSO (Molecular biology grade, Sigma Millipore) to prepare a stock solution at 1.5 mM, then it was aliquoted and kept in the dark at −20 °C. The buffer for hS100A7 was 20 mM Hepes at pH 7.4 and 100 mM NaCl. The buffer for mS100A7 and mS100A7ΔC was 20 mM Hepes at pH 7.4, 100 mM NaCl, 1% glycerol, and 1 mM TCEP. Protein and buffer were preincubated with Chelex resin.

Protein was mixed with an amount of ZP4 to bring the concentration to 2 μM. After a 20 min equilibration, the protein/ZP4 mixture was aliquoted into black Eppendorf tubes, and ZnSO_4_ was added to bring the solution to the desired Zn^2+^ concentration. The mixture was allowed to equilibrate for 60 min prior to measurement. Mixtures and additions were adjusted to ensure each solution ended with the same dilution factor and a final protein concentration of 5 μM (hS100A7, mS100A7) or 3 μM (mS100A7, mS100A7ΔC). The emission spectrum was recorded using a Horiba Jobin Yvon Fluoromax-3 fluorimeter and a submicro fluorometer Starna quartz cuvette. The emission spectrum was recorded from 510 to 530 nm, using 490 nm as excitation wavelength. All experiments were performed at least in triplicate.

The plots and fits for all titrations are shown in Fig. S4. The plot and fit for the representative data that are closest to the mean are shown in Figure 2. For hS100A7, we observed a hyperbolic binding curve as reported previously (15), but the plateau was reached at ∼6 μM *versus* ∼10 μM Zn^2+^. We attribute this small difference to our utilizing reduced *versus* the oxidized protein used previously. The titration curves were hyperbolic for the 5 μM mS100A7 and sigmoidal for both the full-length and truncated protein at 3 μM, with all plateaus at ∼5 μM Zn^2+^. The data were normalized (X-Xmin/(Xmax-Xmin)) and fit by nonlinear least squares regression in DynaFit (36) to standard 1:1 binding equations for both ZP4 and each S1007 protomer. In this model, the two symmetric zinc binding sites in the S100A7 homodimer are assigned the same affinity. To minimize errors, each binding curve was fit individually, then the reported K_d_ apparent was calculated by averaging of the individual values returned for each independent measurement.

### NMR

Samples of ^15^N-enriched mS100A7 at 100 μM were prepared in a buffer containing 20 mM Tris-HCl at pH 8.0, 50 mM NaCl, 1% glycerol, and 1 mM DTT. Experiments were acquired with CaCl_2_ concentrations of 0, 100, 200, 300, and 1000 μM corresponding to mS100A7:Ca^2+^ ratios of 1:0, 1:1, 1:2, 1:3, and 1:10. Samples of ^15^N-enriched hS100A7 were prepared in a buffer containing 20 mM Tris at pH 8.0, 100 mM NaCl, 1 mM TCEP, 2 mM EDTA or 2 mM CaCl_2_, and 10% D_2_O. 2D ^15^N-^1^H heteronuclear single quantum coherence experiments were recorded in 5 mm tubes at 25 °C using a Bruker AVANCE 800 MHz spectrometer equipped with a TCI cryoprobe. The pulse sequence hsqcetfpf3gpsi2 from the standard suite of Bruker pulse programs was used, and all spectra were recorded with 32 scans, 2048 and 128 points in the direct and indirect dimensions, respectively. Data processing and analysis was carried out using TopSpin 3.6, NMRPipe, and CcpNMR (version 2.4.2).

### Crystallization

Before crystallization, the purified protein was diluted in a buffer containing 20 mM Tris at pH 8.0, 100 mM NaCl, 1% glycerol, 1 mM TCEP, and 2 mM CaCl_2_ then concentrated to 12 to 14 mg/ml using a Amicon Ultra-filtration system with 3 kDa MWCO. Initial crystallization screening was undertaken with a Mosquito LCP Robot and four commercial screens (JSCG+, JCSG core suite I and II, and Index HT) (Qiagen). The protein and the crystallization solution were mixed in a 1:1 ratio at 20 °C. Numerous macleod crystals were obtained for condition 95 of Index HT (0.1 M potassium thiocyanate, 30% PEG MME 2K). These crystals could not be reproduced in 24-well Linbro plates; so crystal optimization was carried out in 96-well plates. The crystallization conditions were refined by varying the PEG concentration and by using the additives kit from Hampton research. Crystals with the best diffraction quality were obtained in 0.1 M potassium thiocyanate, 20% PEG MME 2K, and 0.01 M strontium chloride.

### X-ray diffraction and structure determination

The crystals were soaked in the well precipitant supplemented with 20% glycerol for cryoprotection, scooped with a nylon loop, and flash frozen by immersion in liquid nitrogen. The diffraction sets were collected at the Northeastern Collaborative Access Team beamline at the Advanced Photon Source. The indexation, integration, and scaling steps were performed with Xia2 using the XDS and XSCALE pipeline package (37, 38). The structure of mS100A7ΔC was determined by molecular replacement using the automated pipeline MoRDa (39) with 1E8A as the initial model. The models obtained after the phasing were manually edited using COOT (40) and refined using REFMAC5 (41).

## Data availability

The sequences used for this study can be found in the UniProt database with the accession numbers: mS100A7 (S100A15a)(Q6S5I3), hS100A7(P31151). For sequence alignment, the NCBI accession numbers for human homologs are: XP_008062097.1 (*Carlito syrichta*), XP_006177234.1 (*Camelus ferus*), XP_014720465.1 (*Equus asinus*), and XP_004026778.1 (*Gorilla gorilla gorilla*); for murine homologs: XP_055215704.1 (*Gorilla gorilla gorilla*), XP_014720466.1 (*E. asinus*), XP_019286813.1 (*Panthera pardus*), and XP_002686149.1 (*Bos taurus*). The structure of mS100A7 has been deposited at the PDB under PDBID code 8S9W.

## Supporting information

This article contains supporting information.

## Conflict of interest

The authors declare that they have no conflicts of interest with the contents of this article.
