# Supplementary material for: Comparative analysis of the physical properties of murine and human S100A7: Insight into why zinc piracy is mediated by human but not murine S100A7
Source: J Biol Chem. 2023 Sep 26;299(11):105292. doi: 10.1016/j.jbc.2023.105292 (PMC10598741; doi:10.1016/j.jbc.2023.105292)
Supplement: Supporting Tables S1–S3 and Figures S1–S5 [file mmc1.docx]

**Supporting Information**

**Table S1. Dynamic light scattering of mS100A7 in different states of metal loading**

|  | Rh^1^ (nm)  [Polydispersity] Peak 1 | %Mass  Peak 1 | Rh (nm) [Polydispersity] Peak 2 | %Mass  Peak 2 | Rh (nm) [Polydispersity] Peak 3 | %Mass  Peak 3 |
| --- | --- | --- | --- | --- | --- | --- |
| EDTA | 1.37 [27.0%] | 100 | 40.15 [32.9%] | 0 | 119.06 [11.2%] | 0 |
| Ca^2+^ | 1.36 [25.7%] | 99.3 | 28.88 [34.0%] | 0 | 585.08 [50%] | 0.6 |
| Zn^2+^ | 2.39 [9.5%] | 52.8 | 47.41 [12.6%] | 0 | 957.94 [39.1%] | 47.2 |
| Ca^2+^+Zn^2+^ | 2.50 [2.6%] | 33.6 | 182.93 [11.1%] | 0.5 | 1259.11 [29.7%] | 65.9 |

^1^Rh : hydrodynamic radius.

All experiments were performed at room temperature at a protein concentration of 10 µM in 20 mM Tris at pH 7.4, 100 mM NaCl, and either 50 μM EDTA, 50 μM CaCl_2_, 10 μM ZnSO_4_, or 50 μM CaCl_2_ + 10 μM ZnSO_4_.

**Table S2. Interhelical Angles of mS100A7, hS100A7 and a selection of other S100 proteins**

|  | Human S100A7 | Mouse S100A7 | Human S100A11 | Human  S100A12 | Human  S100A12 | Human S100A6 | Human S100A6 | Human S100B | Human S100B |
| --- | --- | --- | --- | --- | --- | --- | --- | --- | --- |
| Metals | Ca + Zn | Ca | Ca | Ca | Apo | Ca | Apo | Ca | Apo |
| PDB | 3PSR | 8S9W | 2LUC (8)^1^ | 1E8A | 2WCE | 1K9K | 1K9P | 2H61 | 2PRU (l1)^1^ |
|  |  |  |  |  |  |  |  |  |  |
| H_I_-H_II_ | 36.8° | 36.7 | 51.5 | 44.7 | 53.7 | 40.7 | 46.8 | 41.3 | 54.9 |
| H_II_-H_III_ | 56.8° | 72.6 | 57 | 61.5 | 35.8 | 66.5 | 36.1 | 79.5 | 23.7 |
| H_III_-H_IV_ | 49.9° | 57.4 | 62.7 | 53.6 | 21.7 | 66.7 | 23.7 | 77.3 | 27.5 |
| H_I_-H_IV_ | 51.9° | 50.4 | 48.5 | 55.8 | 67.3 | 47.0 | 65.1 | 49.3 | 67.1 |
| H_I_-H_I’_ | 34.2° | 50.4 | 24.2 | 26.3 | 37.8 | 29.1 | 41.0 | 30.3 | 31 |
| H_IV_-H_IV_’ | 34.3° | 52.1 | 33.4 | 38.2 | 18.1 | 36.2 | 40.9 | 34.6 | 21.7 |
| H_I_-H_IV’_ | 63.6° | 70.5 | 57.2 | 63.9 | 72.4 | 57.4 | 72.5 | 62.9 | 73 |

^1^For NMR structural ensembles, the structure closest to the mean was used for the analysis.

Hx refers to chain A and the Hx’ to chain B. Helices were defined using the Axes/Planes/Centroid tool and interhelical angles were measured using Chimera. The helixes extremities were manually determined, then Chimera was used to determine the interhelical angles between the selected helixes. The major differences between the human and mouse protein are highlighted in yellow. The helixes were defined as:

3PSR: H_I_: Gln4-Tyr19; H_II_: Lys28-Phe39; H_III_: Tyr53-Lys61; H_IV_: Phe71-His90

8S9W: H_I_: Pro5-Ala2; H_II_: Leu32-Ser 42; H_III_: Ln54-Asp66; H_IV_: Phe75-Leu97

2LUC: H_I_: Glu9-Ala25; H_II_: Ly36-Leu47; H_III_: Pro57-Leu67; H_IV_: Phe77-Val99

1E8A: H_I_: Thr1-Arg20; H_II_: Ser28-Leu40; H_III_: Asp49-Asp61; H_IV_: Phe70-His87

2WCE: H_I_: Thr1-Val 19; H_II_: Lys29-Glu39; H_III_: Asp49-Asn63; H_IV_: Asp69-Ala84

1K9K: H_I_: Pro4-Gly21; H_II_: Ser30-Leu42; H_III_: Gln49-Asp61; H_IV_: Phe70-Lys89

1K9P: H_I_: Cys3-Gly21; H_II_: Lys31-Leu42; H_III_: Ala51-Asn63; H_IV_: Phe70-Tyr84

2H61: H_I_: Ser1-Gly19; H_II_: Lys28-Leu40; H_III_: Glu49-Asp61; H_IV_: Asp69-Glu89

2PRU: H_I_: Ser1-Tyr17; H_II_: Lys28-Glu39; H_III_: Lys48-Gly64; H_IV_: Asp69-Thr82

**Table S3. Crystallographic and refinement statistics for the structure of Ca^2+^-loaded mS100A7ΔC.**

| **Data Processing** |  |
| --- | --- |
| Wavelength | 0.91 |
| Space group | P2_1_2_1_2_1_ |
| a, b, c (Å) | 31.54, 68.40, 85.61 |
| α, β, γ (°) | 90, 90, 90 |
| Resolution (Å) | 34.16-1.69 (1.72- 1.69) |
| Number of observed reflections | 278958 (13172) |
| Number of unique reflections | 21457 (1044) |
| Redundancy | 13 (12.66) |
| Completness (%) | 98.24 (92.94) |
| R_merge_ | 0.07 ( 1.998) |
| I/σ | 15.7 (1.5) |
| CC_1/2_ | 99.9 (80.4) |
| **Refinement statistics** |  |
| R_work_/R_free_ (%) | 0.186 (0.236) |
| Number of atoms | 1685 |
| Protein | 1568 |
| Calcium ion | 4 |
| Sodium ion | 2 |
| Buffer molecules | 31 |
| Water | 80 |
| **B-factors** |  |
| Protein | 42.80 |
| Calcium ion | 34.75 |
| Sodium ion | 40.55 |
| Water | 48.94 |
| Buffer molecules | 60.68 |
| **Root mean square deviation** |  |
| Bond length (Å) | 0.018 |
| Bond angles (°) | 1.96 |
|  |  |

Buffer molecules refers to acetate ions, thiocyanate ions and glycerol molecules.

**Figure S1. Thermal denaturation of mS100A7 monitored by circular dichroism.** The experiment was performed at a protein concentration of 10 µM in a buffer containing 20 mM Hepes at pH 7.4, 50 mM NaCl, 1% glycerol and 1 mM TCEP. The plot shows change in CD at 222 nm. The change in CD does not reach a plateau at 110 °C indicating that the protein is not totally denatured even at this high temperature.


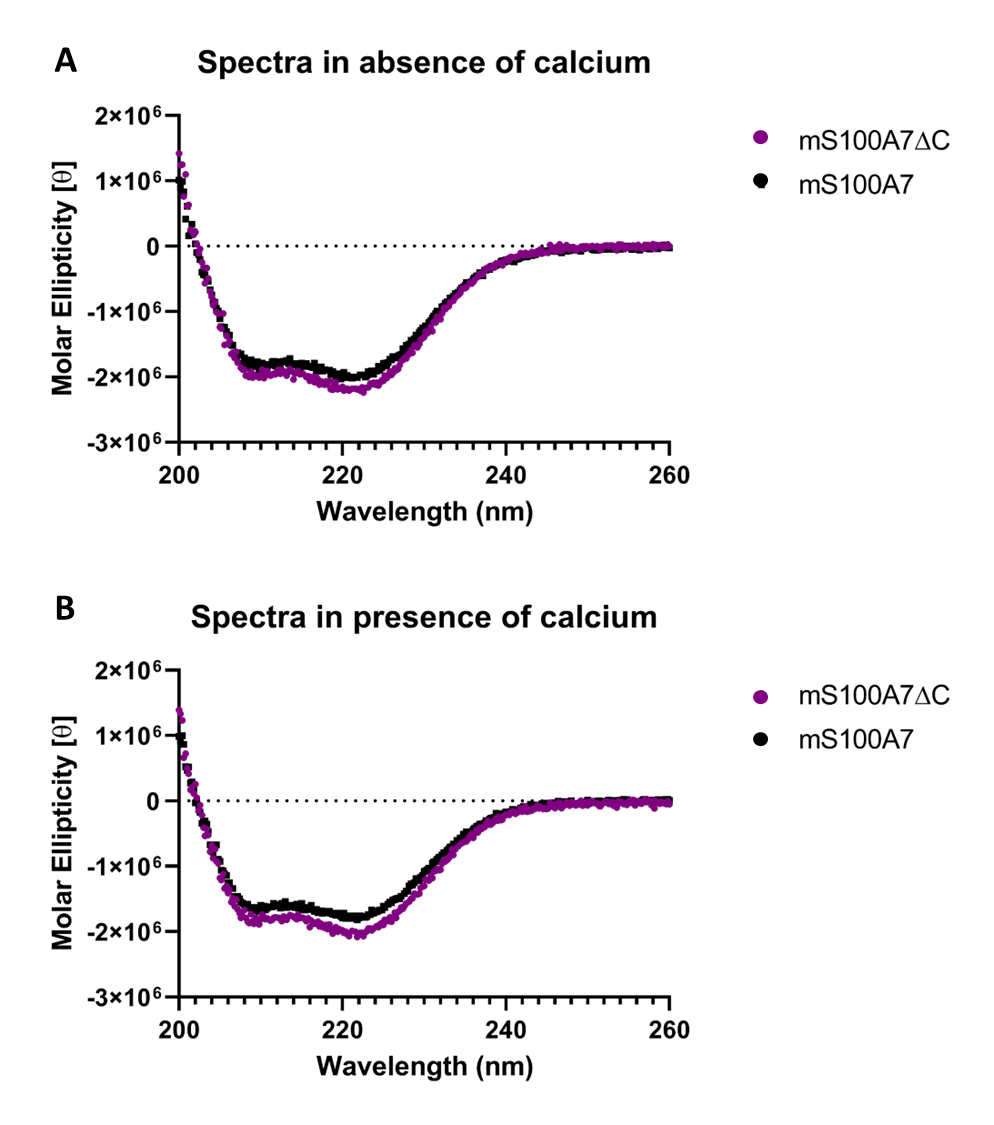


**Figure S2. Comparison by circular dichroism of mS100A7wt and mS100A7ΔC.** All the experiments were performed at room temperature with a protein concentration of 10 µM in a buffer containing 20 mM Hepes at pH 7.4, 50 mM NaCl, 1% glycerol and 1 mM TCEP. (A) Spectra acquired in the absence of Ca^2+^. The protein was treated using Chelex resin to ensure the absence of cations. (B) Spectra acquired with 100 µM Ca^2+^ in the buffer.


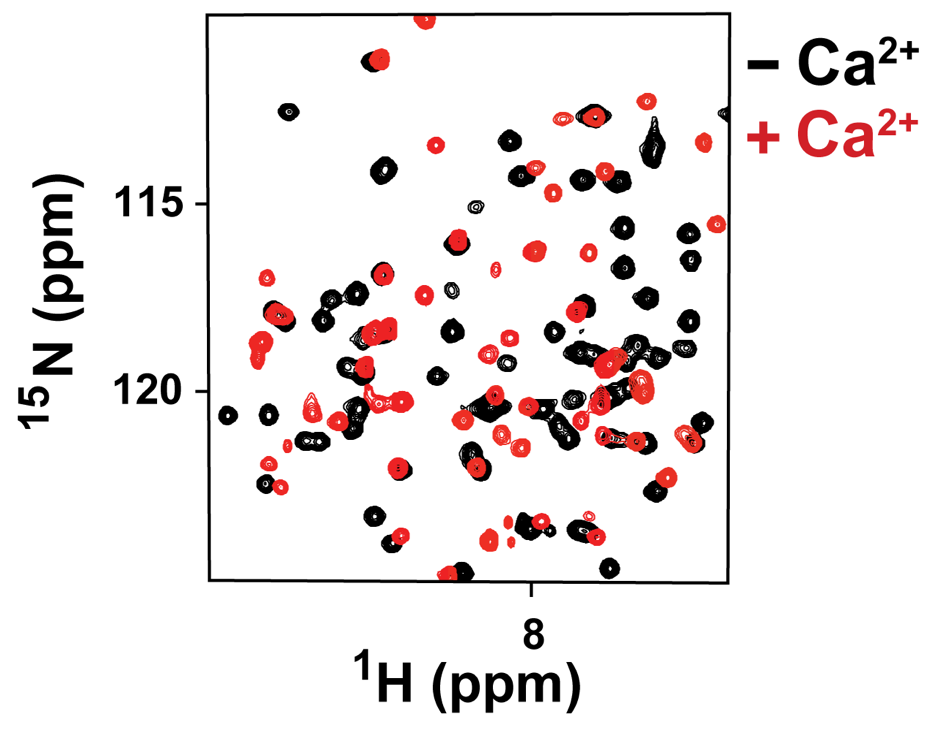


**Figure S3. NMR chemical shift changes reflecting the conformational change induced in human S100A7 by the binding of Ca^2+^.** Zoomed in view of the central region of the 800 MHz ^15^N-^1^H HSQC spectrum of 150 μM hS100A7 of hS100A7 in the absence (black) and presence (red) of Ca^2+^. The spectra were recorded at 25 °C in a buffer containing 20 mM Tris at pH 8.0, 100 mM NaCl, 1 mM TCEP, 2 mM EDTA or 2 mM CaCl_2_, and 10% D_2_O. The large number of changes in the signals (i.e., lack of overlap) is characteristic of the Ca^2+^-induced change in S100 proteins, arising primary from the reorganization in the positioning of helix III with respect to the rest of the protein.


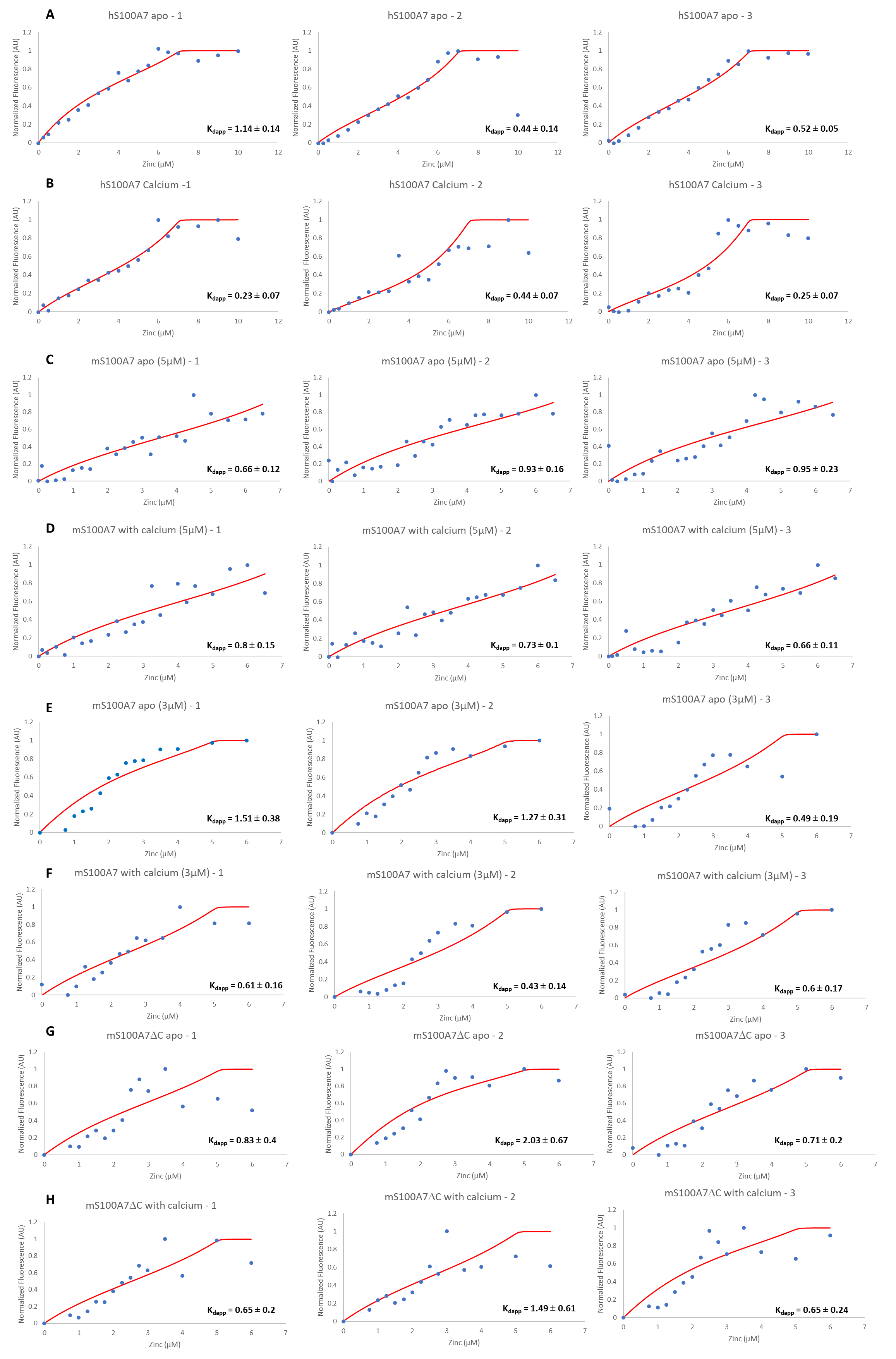


**Figure S4. Individual plots and fitting for the ZP4 titration with hS100A7, mS100A7 and mS100A7ΔC.** (A, B) Titrations performed with 5 μM hS100A7 in the absence and presence of 25 μM Ca^2+^. (C, D) Titrations performed with 5 μM mS100A7 in the absence and presence of 25 μM Ca^2+^. (E, F) Titrations performed with 3 μM of mS100A7 in the absence and presence of 15 μM Ca^2+^. (G, H) Titrations performed with 3 μM mS100A7ΔC in the absence and presence of 15 μM Ca^2+^. The calculated apparent K_d_ values are listed in the bottom right of each plot. All data were fit with two equivalent binding sites per dimer model. The red line shows the fit to the data calculated in DynaFit. The plots shown in **Figure 2** are: (A) hS100A7apo- 3, (B) hS100A7-calcium- 1, (C) mS100A7 apo (5µM)- 2, (D) mS100A7 with calcium(5µM)- 2, (E) mS100A7 apo (3µM)- 2, (F) mS100A7 with calcium (3µM)- 1, (G) mS100A7ΔC apo- 3, and (H) mS100A7ΔC with calcium- 1.


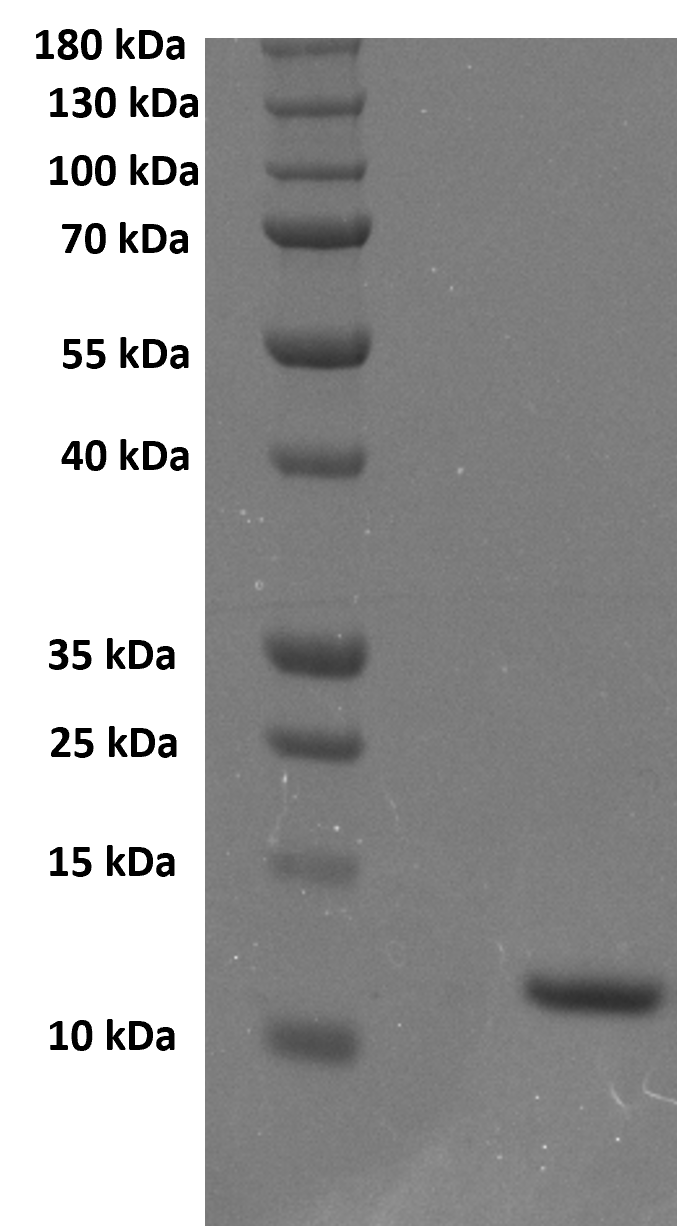


**Figure S5. Zn^2+^-loaded mS100A7 is not disulfide cross-linked.** SDS-PAGE gel run in the absence of reducing agents showing that the protein runs at the mass of one protomer and therefore not cross-linked.
